# Recognizing pain as an early warning symptom of ischemic cardiovascular disease: A qualitative artistic representation of the journey

**DOI:** 10.1080/24740527.2020.1801339

**Published:** 2020-09-24

**Authors:** Sheila O’Keefe-McCarthy, Karyn Taplay, Allison Flynn-Bowman, Lisa Keeping-Burke, Vanessa Sjaarda, Lynn McCleary, Jean Abernethy, Melanie Prentice, Kayleigh Tyrer, Jenn Salfi

**Affiliations:** a Faculty of Applied Health Sciences/Department of Nursing, Brock University, St Catharines, Ontario, Canada; b Department of Nursing and Health Sciences, University of New Brunswick, Saint John, New Brunswick, Canada

**Keywords:** prodromal ischemic cardiac pain, adults, qualitative, arts-based research

## Abstract

**Background**: Understanding the experience of prodromal ischemic cardiac pain and associated symptoms through use of literary and visual art evokes heightened a wareness of the emotional journey.

**Aims:**

The aim of this study was to describe the initial early prodromal pain-related symptoms and feelings associated with adjusting to this new cardiac health concern and explore the subjective experience of coming to the realization and awareness of developing heart disease.

**Materials and Methods:**

This study is a secondary supplemental qualitative analysis, using an arts-based embodied layered exploration assisted to translate the experiences of 23 individuals’ journeys through symptom recognition. The analytic process involved three iterative layers: qualitative descriptive analysis of participant pain narratives, interpretation with thematic poetry, and representation via visual art to evoke an aesthetic, heightened level of understanding of the data.

**Results:**

Denial and disbelief, encroaching pain and symptoms of heart disease, and self-recrimination were three themes that emerged from the data. Pain described by participants brought forward the emotional dimensions of the experience. Participants described their process of realization as a tumultuous time, fraught with feelings of vulnerability and uncertainty, where anger and self-effacing ridicule permeated their thoughts that were tempered with profound gratitude at survival.

**Conclusion:**

Bridging the connection between science and art to disseminate awareness of the nature of living with cardiac-related prodromal pain and disease is novel. Providing invitation and entrance into an individual’s pain experience through qualitative inquiry with use of arts-based approaches makes visible the emotional meaning of pain.

## RÉSUMÉ

**Contexte**: La compréhension de l’expérience de la douleur cardiaque ischémique avant-coureuse et des symptômes qui lui sont associés par l’utilisation de la littérature et des arts visuels permet de mieux prendre conscience du cheminement émotionnel.

**Objectifs**: Cette étude avait pour but de décrire les premiers symptômes avant-coureurs liés à la douleur et les sentiments suscités par l’adaptation à ce nouveau problème de santé cardiaque, ainsi que d’étudier l’expérience subjective de la prise de conscience d’être en train de déveloper une maladie cardiaque.

**Matériel et méthodes**: Cette étude constitue une analyse qualitative supplémentaire secondaire utilisant une méthode d’exploration par les arts à plusieurs niveaux pour traduire le cheminement de 23 personnes en ce qui concerne la reconnaissance des symptômes. Le processus d’analyse comportait trois niveaux itératifs : une analyse descriptive qualitative des récits sur la douleur des participants, l’interprétation par la poésie thématique et la représentation par l’art visuel, afin de susciter un niveau de compréhension esthétique et plus élevé des données.

**Résultats**: Le déni, l’incrédulité, la douleur envahissante et les symptômes de maladie cardiaque, de même que l’autorécrimination, sont trois des thèmes qui sont ressortis des données. La description que les participants ont faite de la douleur a révélé les dimensions émotionnelles de l’expérience. Les participants ont décrit leur processus de prise de conscience comme une période tumultueuse, marquée par la vulnérabilité et l’incertitude, où la colère et une dérision discrète imprégnaient leurs pensées, lesquelles étaient tempérées par une profonde gratitude pour avoir survécu.

**Conclusion**: L’établissement d’un lien entre la science et l’art pour faire connaître la vie avec la douleur avant-coureuse et la maladie cardiaque consistue une approche novatrice. Le fait d’être invité à pénétrer à l’intérieur de l’expérience de la douleur vécue par un individu par le truchement d’une enquête qualitative ayant recours à des approches axées sur les arts rend visible la signification émotionnelle de la douleur.

## Introduction

Globally, people fail to recognize the early warning symptoms (prodromal symptoms, PS) indicative of the development of ischemic coronary artery disease (CAD). CAD affects over 2.4 million Canadians and is the second leading cause of morbidity and mortality.^1,2^ A recent report issued by the Heart and Stroke Foundation of Canada warns Canadians that every 20 minutes a person will die of a fatal acute coronary syndrome (ACS) event due to not recognizing the symptoms of heart disease.^1^ The economic burden of ACS is considerable in Canada and this year has reached Canadian $28.3 billion related to physician services, hospital costs, lost wages, and decreased productivity.^2,3^ Approximately 70,000 acute myocardial infarctions occur each year, and approximately 14,000 Canadians will die from ACS.^2,3^ Of individuals who survive ACS, 7.7% are readmitted with a second heart attack and 12.5% with unstable angina within one year of an initial hospitalization.^4^ Canadians miss an estimated 1.3 million days of work each year related to the effects of poor health associated with ACS.^4^

Pain experienced from ACS (unstable angina and myocardial infarction) is typically reported as being in the severe pain range (i.e., Numeric Rating Scale ≥ 7/10).^5^ Ischemic pain arising from ACS, whether experienced in the prodromal (preclinical) or acute phase, has been described as chest pain/discomfort, tightness, or heaviness.^6–9^ Others may present with individual, nontraditional prodromal or acute symptoms regarded as “anginal equivalent symptoms” such as fatigue, dizziness, nausea and vomiting, and/or shortness of breath.^2,3,5,10–12^

Prodromal symptoms are specific and nonspecific symptoms that individuals may experience singularly or in clusters, days, weeks, or months prior to a cardiac event, with varying degree of intensity and frequency that dissipate once the event has resolved.^8,13–16^ Incidence ranges from 49% to 92% in men and women.^16^ The most prevalent cardiac-related PS reported by individuals across 28 studies were chest pain (49%), fatigue, (37%) and shortness of breath (25%).^13^

Early recognition of the ischemic warning symptoms is critical to prevent myocardial death and provide immediate reperfusion therapies for obstructive CAD.^1^ Early pain detection and treatment help to circumvent transition from acute cardiovascular pain to forms of persistent pain and to decrease CAD-related morbidity and mortality.^17,18^ Though there have been increased attempts to create awareness and educate about the signs and symptoms associated with heart disease,^1^ the importance of recognition and meaningful uptake of this knowledge through artistic, creative, and concrete ways has been minimal in knowledge mobilization and transfer strategies.^19,20^

Pain is a subjective journey. It is complex to understand and is multidimensional in nature. Pain experienced is a product of a dynamic interplay of sensory–discriminative, cognitive–evaluative, and affective–motivational components, unique to each person.^21,22^ Individuals’ pain narratives contain key words that stress pain symptom qualities, draw our attention to specific timing and severity of symptoms, and expose individuals’ emotions, feelings, and thoughts experienced during the time of illness.^9^ Pain is experienced within the context of an individual’s life; it disrupts. Pain infiltrates every aspect of a person’s being. It has been argued by Charon^23^ that the affective–emotional dimensions of an illness experience may be obscured, should clinicians practice from the dominant biophysical medical model. Where the human dimensions of a health-related illness experience may be perceived as lower in priority than the often necessary technological or specialized focus of health care or treatment regimen.^23^

Pain narratives of the journey through recognition of the early prodromal pain-related symptoms are a mechanism to help people articulate their pain stories. Schwind^19^ asserts the importance of actively listening and understanding illness through patients’ narratives. She argues that illness narratives “are multidimensional, temporally contextualized within internal and external landscapes and always exist within a relationship.”^19(p128)^ Our research team asserts that there is need to create a place and space to bridge art and science together, to revisit and make visible the emotional dimensions of individuals’ cardiovascular pain experiences. To do this, we use an arts-based integrative approach that combines the strengths of qualitative inquiry with a humanistic health care lens.^24–26^ This enables us to create new knowledge by transforming patients’ pain narratives into poetry and art, which represent the emotional dimensions of their pain-related experiences.^26^

## Arts-informed Approaches

Arts-informed approaches have been used to examine the experience of illness and are robust methods to conduct research and disseminate knowledge. This kind of approach permits the use of art at any time along the research trajectory and may include analysis, interpretation, and dissemination.^27–29^ As either a stand-alone qualitative method or an adjuvant tool, arts-informed methods enhance information, knowledge production, and representation.^25,26,30,31^ The purpose of an arts-informed approach is to evocatively translate knowledge, layering or building upon different artistic genres (literary, artistic, and performative representations) to provide what we describe as an arts-based embodied layered exploration (ABELE): an approach to represent a health-related experience. The greatest advantage of using the ABELE approach is the ability to reach relevant and diverse audiences.^25–27^ In this way, arts-informed analysis and translation of a human pain-related experience helps to challenge preconceived notions about pain and enhance understanding of this human condition.^25^

Examining pain with the use of qualitative inquiry not only permits the human voice to be heard as patients’ perceptions and pain experiences are expressed but, more so, it has the ability to humanize clinical practice.^24^ Qualitative research provides health care practitioners the ability to enter into the interiority of a lived health condition or illness experience. Todres et al.^24^ claim that there is a strong relationship between a humanizing value framework for health care and the practice of qualitative research. They posit that dimensions of health care are provided by clinicians along a continuum of humanizing or dehumanizing possibilities. Regarding pain, for instance, viewing the pain experience from the patient’s point of view, rather than from an objective or outsider perspective, emphasizes a form of humanization that gives equal consideration to the patient’s pain experience.^24^ Seeking to understand the impact that pain has on the person by valuing the patient’s subjective authority of that felt and lived pain experience validates the patient’s feelings, moods, and emotions and thus brings forth more of a humanizing interaction where the whole person is accepted rather than diminished.^24^

Social theory is often threaded throughout arts-based levels of inquiry, formally or informally.^26^ As new categories or themes emerge, the research team along with the participants and artists collaborate in active knowledge production and, in this sense, co-create new knowledge based on the qualitative data. All work together where the roles are blurred and power differentials are flattened.^26,32^

The use of an arts-informed research to describe the experience of early prodromal pain-related symptoms has not been well explored. Therefore, the purpose of this secondary qualitative analysis, using an arts-informed approach, was to describe the initial early prodromal pain-related symptoms and feelings associated with adjusting to this new cardiac health concern and explore the subjective experience of coming to the realization and awareness of developing heart disease.

## Material and Methods

This secondary qualitative analysis evolved from a primary qualitative descriptive study where our team revised items contained in the Cardiac Prodromal Symptoms Screening Scale (the methods and results are provided elsewhere).^9^ Ethics approval was granted from the Social Sciences Ethics Board at  Brock University (file number 15-325). All participants provided an informed written consent to have their data re-analyzed in a secondary qualitative analysis intended for arts-based translation and dissemination.

For the purposes of this secondary analysis, the emerging themes identified in the parent study required a more in-depth level of analysis. Heaton^33^ describes this as a supplemental secondary qualitative analysis, where the iterative investigative process of the primary study reveals new information, or an unfolding of an issue not completely flushed out that compels re-entry into the data.

### Supplemental Analysis Using an Arts-Based Approach

Using the existing qualitative narratives of the 23 women and men who assisted with the revision of the Prodromal Symptoms Screening Scale^9^ (see Table 1 for participant demographics), our team re-analyzed the transcripts and field notes from individual interviews (*N* = 12) and focus groups (*N* = 4). Our aims were to (1) expand on the integral information and new understandings that emerged from the primary study and (2) advance knowledge with of use evocative methods (artistic mediums) and disseminate the complex, dynamic, and subjective experience of recognition of the early ischemic symptoms of heart disease. Employing an arts-based approach to this supplemental analysis allowed our team to undertake qualitative analysis of participant narratives and apply literary interpretations using thematic poetry. We further represented the poetry through visual art forms to share this knowledge in an evocative manner.^27^

**Table 1.** Demographics of participants.

Acute Coronary Syndrome Participants’ demographics			
		Mean (SD)	Range
Age (years)		65.8 (8.1)	43–78
	Level	Frequency (*n*)	Proportion (%)
Sex	Male	14	60.9
	Female	9	39.1
Ethnicity	White/Caucasian	21	91.3
	Japanese	1	4.3
	South and Southeast Asian	1	4.3
Highest education	Community college/junior college	9	39.1
	Less than high school/secondary school	3	13.0
	High school/secondary school	5	21.7
	University/4-year college	1	4.3
	Postgraduate education/graduate school or higher	5	21.7
Marital status	Married/common-law/cohabitating	16	69.6
	Single	3	13.0
	Divorce/separated	2	8.7
	Widowed	2	8.7
Current employment status	Retired	16	69.6
	Unemployed	5	21.7
	Disability	1	4.3
	Employed full time	1	4.3
Overall household income (Canadian $)	0	1	4.3
	10,000–29,000	3	13.0
	30,000–49,000	5	21.7
	50,000–69,000	5	21.7
	70,000–89,000	2	8.7
	90,000–109,000	2	8.7
	>110,000	5	21.7
Class of angina pectoris	Grade 1	10	43.5
	Grade 2	5	21.7
	Grade 3	1	4.3
	No angina/not applicable	7	30.4

The ABELE approach is an organic, iterative, and artistic integrated approach using qualitative and arts-based methods to fully unravel the complexities that individuals experience along the health–life continuum. For this supplemental analysis we layered qualitative descriptive analysis with various representational forms or mediums of artistic expression to further interpret and represent data. Three creative genres were used to disseminate the data and built upon each other: literary techniques (i.e., poetry), visual art (i.e., paintings), and exhibit and/or performance (i.e., theater).

### Qualitative Description: Layer 1

The intent of employing qualitative description is to derive rich participant descriptions of cardiac-specific prodromal symptoms without the influence of the researcher’s perspective or a theoretical lens to guide the inquiry.^34,35^ This approach can result in multilayered meanings from the data by preserving the verbatim illustrative quotes without imposing a narrative or interpretative intentionality on the data. This provides a straight descriptive summary of the informational contents of the data.^34,35^

The current secondary analyzes were conducted by three members of the research team (S.O.M., K.T., A.F.B.). Disagreements (e.g., of wording, coding, or themes) were handled via consensus of all involved. Content analysis assists to summarize the informational content of data.^36,37^ Participant data were coded and organized into respective categories and emerging themes. The team met at various times to discuss, challenge each other, and make decisions to ensure that codes/categories and themes remained grounded in the original data.^38^ Each transcript was analyzed by counting the participant responses and repetitive use of words or metaphors to describe prodromal symptoms and then coded in terms of the evolving themes.^34^

### Layer II

Next the principal investigator created thematic poetry; that is, a poem created from the main themes derived from the qualitative analysis,^39^ with use of the original words, word clusters, and phrases from the participants. Attention focused on the tones, cadences, pauses, and high and low energy points observed in the interviews and captured in the field notes and heard on the audiotapes or read in the transcripts. All of this taken together was reorganized into stanzas to reflect the themes.^39,40^ Our team worked together in interactive cycles of data analysis, immersion, and reflection, all while remaining open to the evolving nature of the data as key meanings and understandings were identified. Two opportunities for member checking were conducted with participants from the original study to confirm the themes and to present the thematic poetry and pieces of art. Participants were presented with the overarching themes and given an opportunity to read and respond to the poems. All expressed that the themes as presented were what they felt and that the poetry resonated with their experiences. In the end, four poems reflecting three themes where chosen as the most representative.

### Layer III

Once team consensus was established, four visual artists were invited to join the team and to a private recitation of the poetry. They were asked to consider creating visual art to depict the themes. See Table 2 for the artist characteristics. The artists then created new art pieces in relationship to the poetry they had heard; six pieces of art resulted. A second round of member checking occurred with the same participants to view the art. The participants described the art depictions as a concrete representation of their journey and noted that they were profoundly moved by the art. They felt that the art resonated with them. The participants were very enthused to have this art displayed in the public domain.

**Table 2.** Characteristics of artists.

Demographic items		Range
Age		23–59
No. of years an artist		10–55
Variable	*N*	%
Sex (female)	4	100
Ethnicity (Canadian)	4	100
Race (Caucasian)	4	100
Artistic mediums		
Charcoal/conté Encaustic Pastel Mixed media Acrylic Water paints Fiber Digital	2 1 2 1 3 3 1 1	25 25 50 25 75 75 25 25
Chronic/pain-related Condition/illness	1	25

### Layer IV

The intent of this layer was to present the data in evocative ways in order to mobilize this knowledge; for example, through an artistic–theatrical performance or as an art exhibit. The activities of this layer were presented for general and academic audiences in the form of an art symposium where attendees were provided a recitation of the poetry and an opportunity to meet and greet the artists. An exhibit of the research and art was featured at the 40th Annual Scientific Meeting of the Canadian Pain Society in April 2019 in Toronto, Ontario, Canada. The results provided herein focus on findings from the ABELE analysis of participants’ journeys with recognizing pain as an early warning symptom of ischemic cardiovascular disease.

#### Results

##### Demographics

Twenty-three participants consisting of men (*N* = 14) and women (*N* = 9) were recruited and ranged from 43 to 78 years of age. Sample demographics are outlined in Table 1. Four focus groups (*N* = 11 participants) and 12 individual interviews were conducted. An accounting of the incidence and description of prodromal symptoms is provided elsewhere.^9^

##### Themes Reflected through Poetry and Art

Our team identified three overarching themes: (1) denial and disbelief, (2) encroaching pain and symptoms of heart disease, and (3) self-recrimination. Four poems were created to convey these themes using participants’ verbatim words and feelings expressed from the qualitative interviews. The four poems were further conceptualized by artists who created six related pieces of art (one watercolor, two pastels, one charcoal sketching, one collage, and one cartoon–digital art) that metaphorically represented participants’ recognition of the early signs of cardiovascular illness. See Table 3 for a description of the thematic poetry and art.

**Table 3.** Thematic poetry and art and description.

Theme	Poem title	Art title and description
Denial/disbelief	“Warning Signs-Interrupt Us”	*Skeleton Heart* White charcoal on black canvas *Hidden Warning Signs* Collage
Encroaching pain and symptoms of heart disease	“Raw Pain”	*Raw Pain* Watercolor: A Women submerged in pain
	“Running on Empty”	*Running on Empty* Pastel on canvas: Deflated balloons
Self recrimination	“Lucky”	*Lucky* Digital art

###### Theme 1. Denial and Disbelief

Participants articulated their journey of coming to the realization that they had symptoms of heart disease and, as such, could no longer ignore them. Such moments of clarity were fraught with denial and active disbelief and participants chose not to acknowledge warning signs or that symptoms experienced could be associated with the development of an unhealthy heart. One participant perceived that the use of denial was the reason he did not believe or recognize the symptoms as a warning: “… My natural instincts for denial, um, and not to address it, allowed that to kind of occur” (Participant 15, male, age 61). For some, the process of recognition was attributed to those “niggling” or repetitive signs that were perceived as creeping up all around one’s awareness, yet elusive enough to be disregarded. Participants discussed that they could no longer use denial as a mechanism to cope. The nature of the pain was persistent and a constant interruption in their lives. Ischemic symptoms over time leading to their heart attack occurred with increasing frequency and intensity. Another participant spoke of the process of realizing that her symptoms were leading to changes in her health:

I woke up with a squeezing chest pressure, a constant pain … not overly painful. I remember thinking, “Oh my god, I’m having a heart attack,” and then thought, “Don’t be silly. No you’re not!” … And I can go back, maybe 9 months from that episode. I had a little bit of heartburn or something, popped a few Tums; it went away. … Then I don’t remember when the second squeezy pain was, but again, it didn’t last very long. Brushed it off. And this—this one was not going away. (Participant 9, female, age 45)

Throughout the interviews, participants ruminated about how they aggressively chose to “push down and away” their symptoms, not wanting to believe that it could mean or lead to anything detrimental. In the discussions, participants, once ready to accept that the warning signs were not there as a “threat” but rather as a form of protection against death, became open to the realization and recognition of the alert. “Warning Signs-Interrupt Us” was written guided by the exact words spoken by participants and lend meaning to our understanding of this complex process in recognizing early painful prodromal warning signs indicative of CAD.

##### Warning Signs-Interrupt Us

It grips you unaware
sometimes in the middle of the night,
Chest PAIN- it wakes you- awake …
AND
It sneaks up on you unaware, unrecognizable signs … I don’t know … perhaps, I don’t want to know …
I roll over.
Persistent- encroaching … getting stronger … maybe Signs.
Niggling awareness - of what?
Hidden- masked- convoluted in some way- mired in the depth of life’s turns and bends-
an unrecognizable THREAT?
It can’t be real … is it my heart? … no I push it down and
away …
Not hearing my body’s signs, they
Creep up- taking hold- taking over- interrupting us.
PAIN signs more often, more pain, taking over, can’t ignore it- what is it?
Wake up! Look at the signs
Know the warnings of my heart.
Wake up! Or you’ll wake up DEAD.
—Sheila O’Keefe-McCarthy

**Figure 1.**
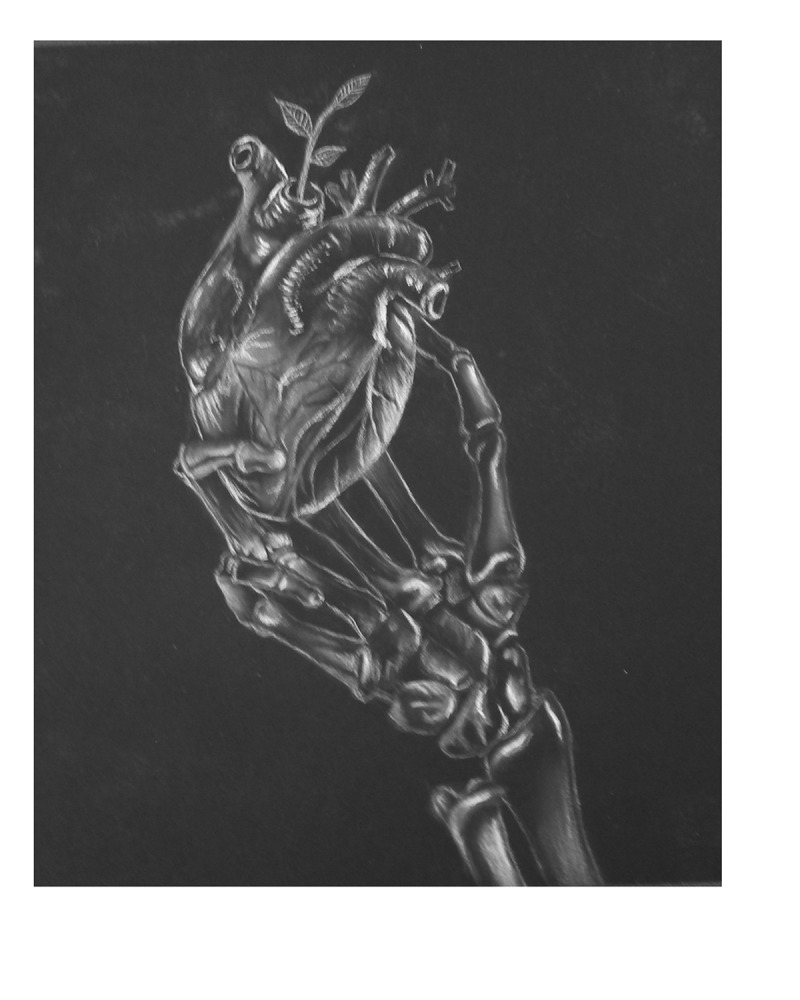
*Skeleton Heart*. Reproduced with permission of the artist, Melanie Prentice.

In response to this poem, one artist was inspired to create a charcoal drawing to further represent the interpretation of warning signs interrupting until they could no longer be ignored. This charcoal drawing by Melanie Prentice is titled *Skeleton Heart* (see Figure 1). The artist found the poem disturbing yet realistic and shared her thoughts of what she was thinking while drawing this depiction.

I found “Warning Signs Interrupt Us” incredibly powerful; it was relatable. I chose to use the white conté and charcoal on dark paper to suggest a ghostly presence. The poem is very chilling; the themes of fear, death, and paranoia are very consistent. In the drawing, the skeletal hand holding the heart could either be interpreted as “death” or as the reader’s [participant’s] own hand. To reflect the last two lines of the poem, “Know the warning signs of my heart. Wake up! Or you’ll wake up DEAD,” I wanted to give a small glimpse of hope into the drawing. A small sprout is depicted rising out. (Artist 1)

The artist’s narrative and metaphoric depiction deepens the viewer’s connection to the participant’s experience, thus opening up multiple ways of understanding the meaning of the original data. It also reflects a journey not only of uncertainty and fear but of hope, as finally recognizing the symptoms permits a way up and out to regain and begin a road to better heart health.

**Figure 2.**
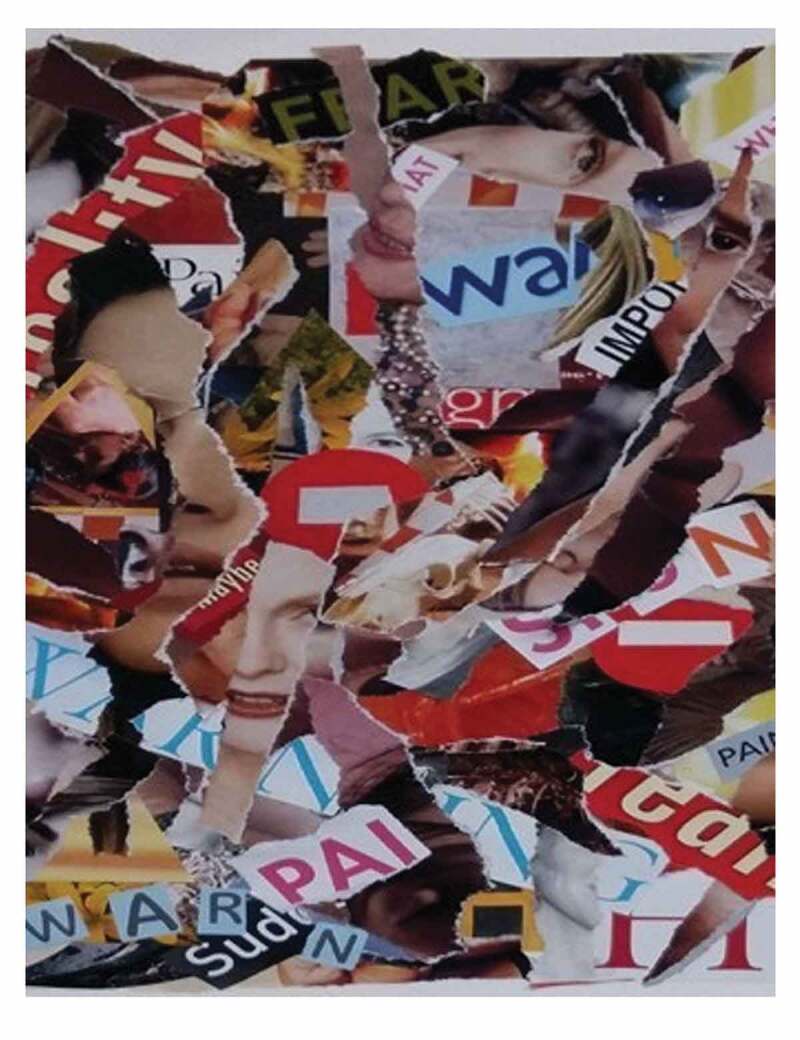
*Hidden Warning Signs*. Reproduced with permission of the artist, Lynn McCleary.

The second artistic piece is a collage created by Lynn McCleary titled *Hidden Warning Signs* (see Figure 2). In this image the artist shares her thoughts of how she set out to capture through images, the movement back and forth she heard in participants’ level of awareness of their symptoms as they rise and recede into and from consciousness. This collage reflects the elusive aspect in the experience of realization. That this experience represents a mixture of trying to grasp that something is different, or something is changing, and yet not having the ability to accurately identify what or where the specific problem lies.

I was struck by the experience of not recognizing warning signs and the body’s warning signs being masked, hidden, or pushed down. I was struck by the phrase “or you’ll wake up dead” so included some images of skeletons or decaying flesh. The process of creating this collage involved creating layers of pieces of women’s bodies, warning signs, masks, and the death images. These images became convoluted by the layering process, with the warning signs more or less obscured or hidden. (Artist 2)

###### Theme 2: Encroaching Pain and Symptoms of Heart Disease

All participants described an array of different early prodromal warning symptoms. The two most frequently mentioned were unexpected cardiac pain and unrecognizable fatigue prior to their adverse cardiac events.

Unexpected cardiac pain was described as coming in waves of cold heat and varied in quality and description. Participant 1 (male, age 74) described chest pain as “Well, it was pain, yes, what number on a scale of 10 … enough that it required me to breathe to control the pain. I felt like it was probably a 7 or 8.” About half of the sample referred to chest pain as “pain, a squeezy or heavy pressure, an uncomfortable constant ache.” Unusual and unexpected chest pain was referred to as “a raw kind of pain” (participant 19, male, age 43), either occurring at rest or with activity. Participants were “surprised” at the kind of pain they experienced and used strong descriptors to explain their pain quality. Another participant described prodromal pain as “gripping, digging, ripping, searing; a burning acid” (Participant 11, female, age 75). For most this kind of pain took one’s breath away and was engulfed with a fear of dying; an unrelenting pain. One participant articulated, “It was frightening! It was so frightening! But it was the most severe pain I’d had” (Participant 3, female, age 73). The insidious and repetitive nature of the pain cycling back and forth, returning more frequently and with increasing severity was described as “a pain that was encroaching upon them” and evoked feelings of vulnerability and of being exposed. This pain experience left participants feeling uncertain and depleted. The poem “RAW PAIN” attempts to capture this subjective, emotional, and painful experience. The participant’s perception lends further insight and helps those of us who are looking from the outside in to feel and hear the emotional experience of prodromal cardiac pain.

#### RAW PAIN

Rising around you like a wave of cold heat … … surprisingly,
unpredictable-unexpected.
Gripping talons of pain …
Ripping, digging in flesh, searing-piercing deep pain
from inside out.
I CAN’T BREATHE …
AM I DYING? … I’M DYING!
A sadistic volcano turned inward erupting- like acid
digging at you … gnawing away,
Stripping me … raw … penetrating, incapacitating pain.
Exposed raw,
Vulnerable …
this unrelenting-PAIN-PAIN.
It is not going away?
Not getting any better … …
—Sheila O’Keefe-McCarthy

**Figure 3.**
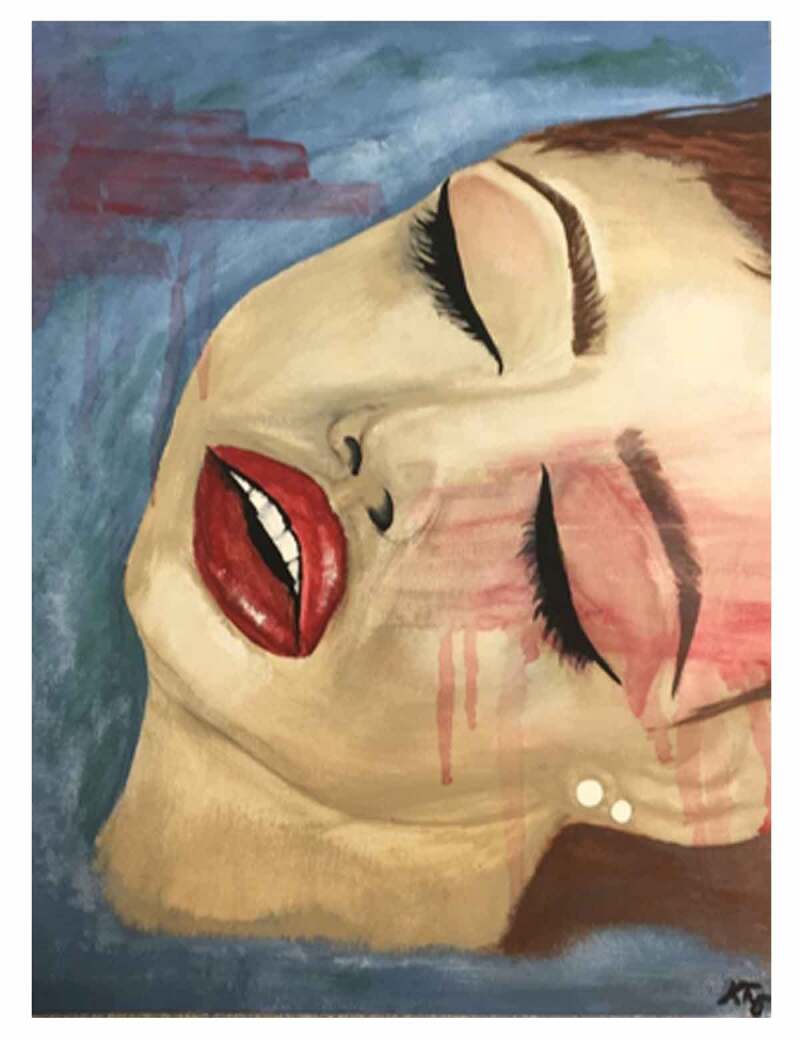
*Raw Pain*. Reproduced with permission of the artist, Kayleigh Tyrer.

“RAW PAIN” was artistically represented through a watercolor painting by Kayleigh Tyrer (see Figure 3) of a woman in the throws of experiencing this prodromal warning. The artist explains:

The blue signifies the coolness of pain. She is floating in the pain that surrounds her. The red strips indicate the pain is stripping away at her. The red drips that run down represent the heat of the pain slowly taking over her body. Her eyes are closed because she is all consumed with the raw pain, she does not know if, or when it will end. (Artist 4)

Unrecognizable fatigue

Unprovoked, unrecognizable prodromal fatigue was described by every participant. When discussed, fatigue was one of the most consistent symptoms, yet was disregarded as irrelevant and not thought of as associated with the development of obstructive heart disease. The interplay between pain and fatigue is known but the experience of this initially did not lead participants to recognition of a serious health issue. One participant shared: “I never thought anything. I thought I was just tired … I would just wear myself out … and try to catch up on sleep if I could. I could sit on the ground and catch 15 minutes of sleep at any time because I was always so tired” (Participant 19, male, age 43). Most attributed fatigue to normal aging or related to other conditions.

Characteristic of this fatigue was that it occurred at the most unexpected times. One man related:

Yeah it was unusual fatigue. I had this probably for 6 months ahead and it was when I wasn’t active. But I couldn’t figure that out. Didn’t make sense to me because all I am doing is talking on the phone. Why should I be getting so tired? … It’s not fatigue from exertion, it’s fatigue from, from total relaxation. You know, because that’s significantly different than what I ever expected. (Participant 5, male, age 74)

This state of utter exhaustion participants spoke about in combination with cardiac prodromal pain was prevalent across all of the interviews. Participants experienced ongoing fatigue and retrospectively identified that they had made accommodations in their everyday life to cope or deal with their tiredness that affected ordinary activities of daily life. Participants expressed that over time their fatigue eventually alerted them that something very serious was happening physically in their body. The poem “Running on Empty” attempts to make sense of the personal experience, to humanize and make meaning of participants’ descriptions of prodromal fatigue.

##### Running on Empty

Sitting tired from lack of activity …
wondering **who** this person is occupying my body space?
Foreign-an insidious, creeping battery drain of unrecognizable … intensity- leading up to … .?
Sort of an emptiness, I … can’t … .
like, my battery was … my endurance to endure was waning … gone.
What am I thinking … I never thought anything … .Always SO tired.
The way I was tired, it wasn’t the way that I should be tired???
Exhausted, deplete of anything; this-is-not-me!
Insurmountable mountains-Oh God-
Stairs, a hill, no I can’t, so I don’t.
Desperately seeking out that slow walking person in front of me, to
blend in, not drawing attention to my lacking … can’t walk, fear seeps in my mind, out my pores.
Why should I be getting tired when I have just started my day?
Not typical fatigue from exertion.
It’s fatigue from total rest …
Different than I ever expected.
Tricked …
Siphoned dry
Stopped in my tracks.
**WHY?**
—Sheila O’Keefe-McCarthy

**Figure 4.**
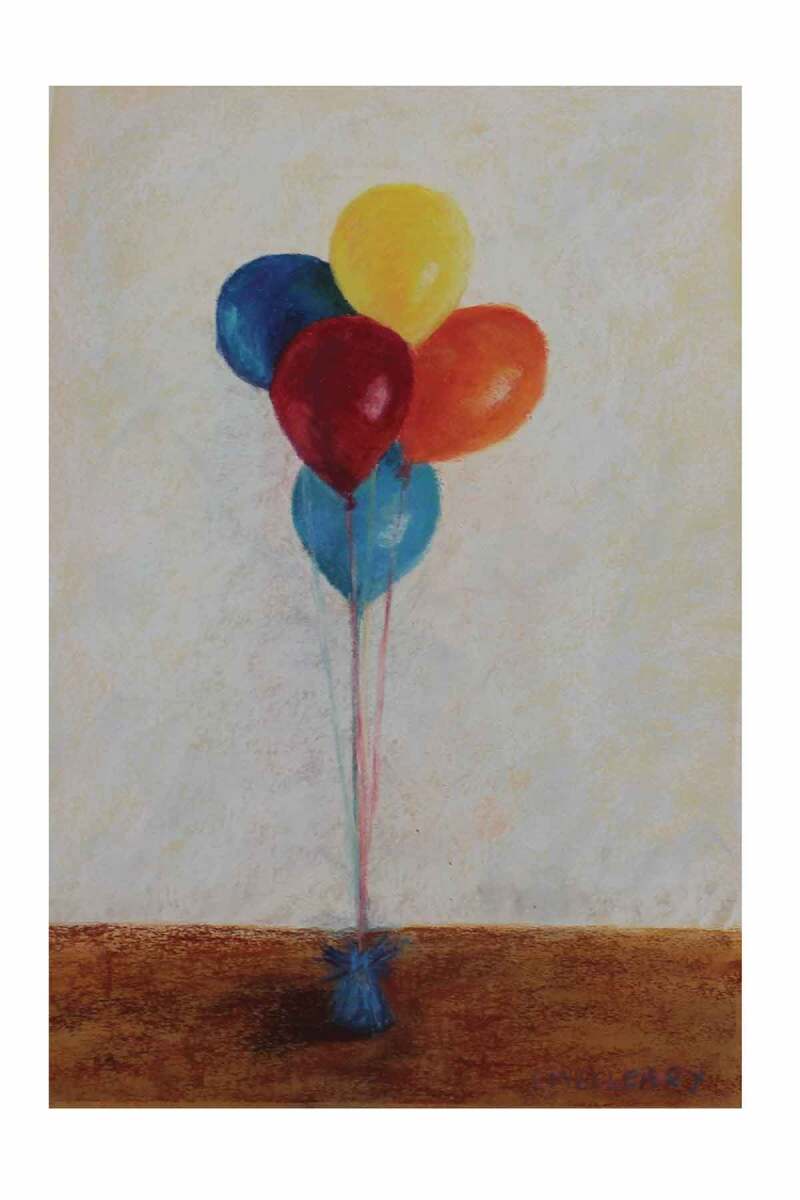
*Running on Empty*. Reproduced with permission of the artist, Lynn McCleary.

**Figure 5.**
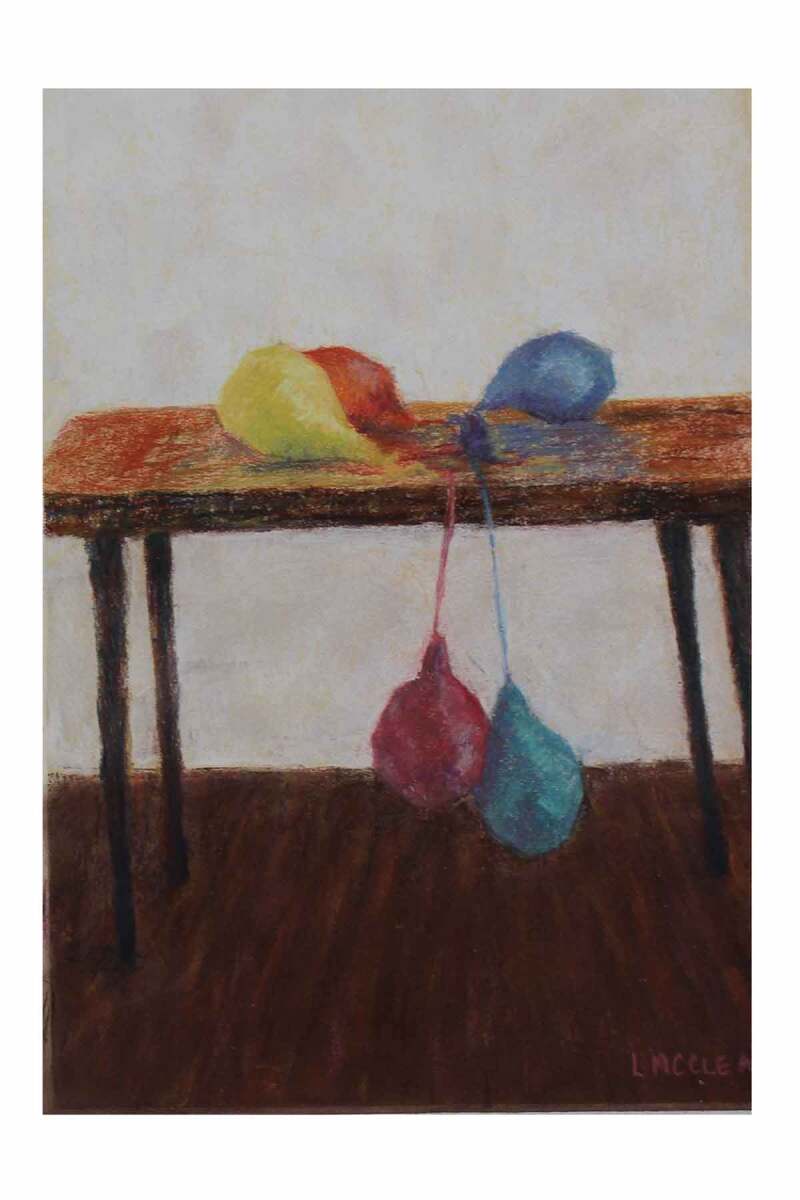
*Running on Empty*. Reproduced with permission of the artist, Lynn McCleary.

Fatigue was experienced at rest, without exertion, a state of exhaustion that was artistically depicted in two pastel drawings by Lynn McCleary (see Figures 4 and 5). These two pastel creations are titled *Running on Empty* and depict the slow, insidious energy drain that participants described. The artist explains her thought process while crafting these.

These are pastel on pastel paper. Reading “Running on Empty” and the abnormal tiredness, I had the image of deflating balloons. I originally planned for a series of three paintings, ending with completely deflated balloons but decided against that. The other poems include gratitude for not ending up dead, so “dead” balloons didn’t seem to fit with the poem’s description of past fatigue that presumably has been overcome. (Artist 2)

###### Theme 3. Self-Recrimination

The experience of coming to the realization that ischemic prodromal symptoms meant the development of CAD (whether recognition occurred leading up to, during, or after a heart attack) prompted all participants to express irritation with themselves. Participants described retrospectively feeling foolish that they had not recognized their symptoms as cardiac in nature. One participant shared these feelings, “I mean, Don’t ignore it. Don’t think you’re stupid. I regret not knowing my family history. I had aching arms and legs, heartburn, I was tired and short of breath. I mean, I had it all and I can’t believe I didn’t know [laughs]” (Participant 9, female, age 45).

Most participants spoke of their gratitude of “missing a bullet” and that they were so happy that they did not come to a fatal end, that they were given a second chance at life. The journey of recognition was a gradual awakening to the reality that some physical change had happened. Awareness was described as an intense process that slowly emerged wherein participants experienced a myriad of emotions in response to their pain-related symptoms.

The poem “Lucky” describes an individual navigating this experience and provides a heightened understanding of the complex experience of eventual pain-related symptom recognition.

##### Lucky

Achy arms and burning legs, pain in my jaw and my back,
Who I, not I,
Why didn’t I think I was having a heart attack?
No drama of grabbing or clutching my chest, nor falling
or fainting on to the floor
as I’ve seen so many times on … my TV before.
I had no chest pain
No signs or symptoms at all-
Only tiredness, some sweating and slight heartburn at night fall.
I never really thought, as perplexed as I was
What all of these signs meant- what it was that gave me great
pause …
I never dreamt of having heart problems, it never entered my head.
Although- now late …
Knowing what I know, I am lucky, so lucky that
I didn’t end up
– DEAD –
—Sheila O’Keefe-McCarthy

**Figure 6.**
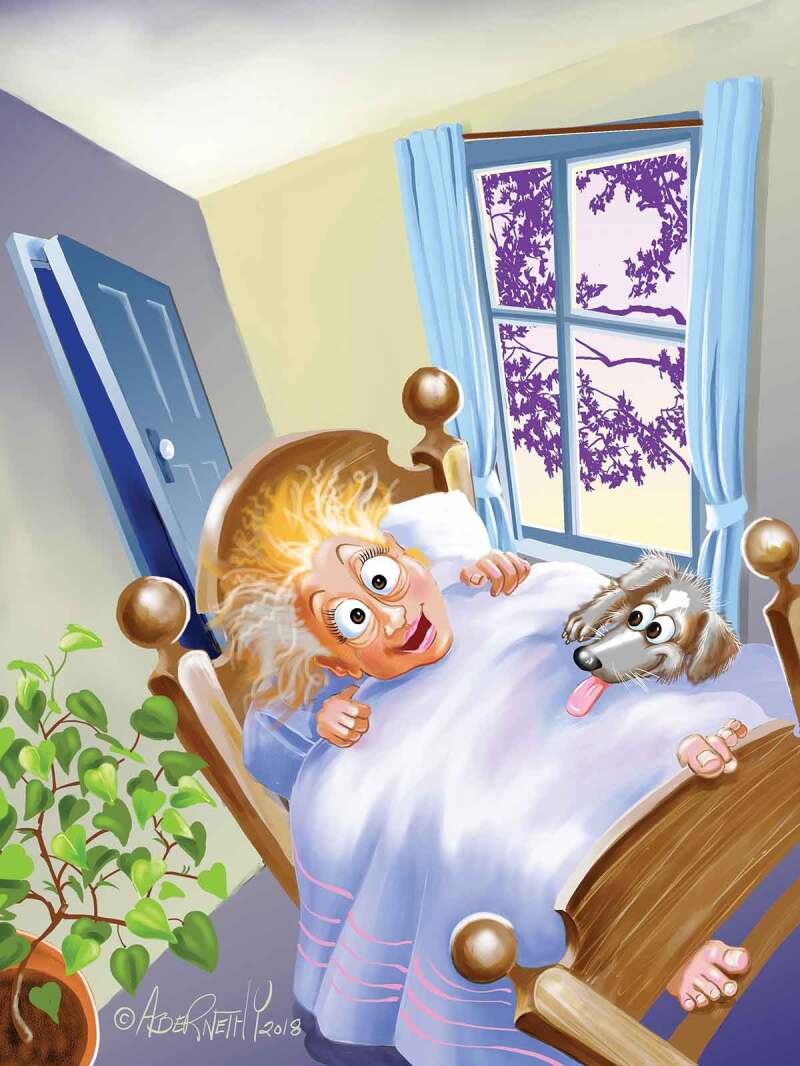
*Lucky*. Reproduced with permission of the artist, Jean Abernethy.

A cartoon drawing was created by Jean Abernethy after listening to a recitation of the poem “Lucky” (see Figure 6). The cartoon created digitally speaks to the underlying use of humor that some participants employed to help them deal with their lack of timely recognition of heart-related ischemic pain and the development of cardiac disease. Interwoven in their words were feelings of deep gratitude that they did not succumb to death but rather lived to see another day; they were laughing at themselves and felt lucky to be alive. The artist comments on the context that helped create this illustration.

This patient’s world has gone wonky! What she once thought, no longer applies! She gets to see another day, sunrise, green growing things … .and her doggie … . She is LUCKY! (Artist 3)

#### Discussion

To our knowledge this is one of the few studies to incorporate an arts-informed integrated approach to analysis, interpretation, and knowledge exchange through use of poetry based on verbatim quotes that were then transformed by artists into visual art. This approach offers new forms of evidence and dissemination to describe pain within a cardiovascular context. Participants describe that experiencing pain, understanding what it could mean, and realizing the need to heed a warning that health has become uncertain or unstable is an emotional journey. This study contributes to qualitative pain inquiry through the use of arts-based approaches to represent the perspective of the acutely ill cardiovascular patient. By transforming participants’ narratives using arts-based translation we are given a unique view of the individual subjective pain experience. Purposefully, we built on aesthetic experience as a method of knowledge dissemination^41^ and created an arts-based exploration of cardiovascular pain—an early warning sign of ischemic disease. The participants’ experiences of journeying through prodromal cardiac pain and associated symptoms indicative of CAD revealed themes of denial and disbelief, encroaching pain and symptoms of heart disease, and self-recrimination.

Active denial of early warning symptoms and disbelieving that prodromal pain and other associated symptoms were indicators of declining cardiac health was articulated by all participants. This has been corroborated by others.^8,14–16^ Psychologist Dr. Stephen Parker, a heart attack survivor, identified that patients experience many differing emotions before, during, and after a cardiac event, including disbelief and anger that it occurred and extreme vulnerability and fear of what the future may bring.^42^ Denial or ignoring the occurrence of the early prodromal symptoms has also been identified by others as problematic.^8,14–16^ Robinson^43^ reported that it is typical for cardiovascular patients to use denial as a coping mechanism, especially in the stages leading up to confirmation of the diagnosis. Use of avoidant coping styles such as active denial is positively correlated to longer decision times to seek aggressive treatment even when chest pain intensity is in the moderate to severe range.^44^ Lack of knowledge of the possible symptoms to be aware of in combination with denial serves as a barrier to prompt recognition and identification of serious warning signs. This perpetuates delay in seeking medical attention early enough to circumvent preventable pain-related cardiovascular morbidity and mortality.^15,44–48^

Arts-based research weaves together the benefits of art and science. Both attempt to illuminate aspects of the human condition with roots in in-depth inquiry, exploration and examination, revelation, and re-presentation to enhance individual understanding of a phenomena.^27^ Merging tenets of creative arts with scientific examination enables answering clinically important research questions in a holistic way. The totality of the internal subjective experience of pain is hard to express, let alone capture with the use of objective biomedical measures typically used in contemporary clinical practice. An arts-based encounter pushes beyond the boundary of accepted kinds of knowledge by evoking the senses through the esthetic, thereby shifting viewers’ perspectives of what they thought they knew about pain and how it is experienced. It challenges the expected notions around pain where preconceived ideas may prevent an understanding of the unique individual before us. Beyond identification and description of the original qualitative themes, arts-informed analysis and dissemination permitted our team to represent the early prodromal pain and illness experience by heightening the viewer’s senses. Drawing on this aesthetic quality to impart research findings is integral to effective knowledge translation and mobilization. Encountering an illness and/or pain lived experience in an artistic way lends to a richer, more meaningful connection and understanding of another person’s pain-related experience. This in turn creates an empathic and compassionate awareness external to one’s self.

Others have used arts-based methods to reveal the experiential meanings in pain and enabled co-created levels of understanding to occur. Padfield’s^49^ work, examining the use of co-created images in the assessment of chronic pain in clinic visits, argues that photographs provide an alternative visual language to communicate pain^49^ and its ambiguity can also encourage emotional expression and personal disclosure^50^ that might not otherwise have occurred. Moreover, use of visual imagery creates a collaborative approach to dialogue within the consulting room.^49^ Utilizing art as an effective medium to discuss and understand the pain experienced may therefore be useful in practice. It deconstructs what Kenny^51^ refers to as the tendency of both clinician and patient to speak but not to listen to each other. Making visible the often unspoken emotional aspects of living with pain represented through literary and visual artistic depictions as in the current study is the critical reason to employ arts-based research: to make visible the emotional meaning of the pain experience.

Photographic images were used in an earlier study where chronic pain was described by 27 participants through use of photovoice. Investigators Baker and Wang^52^ found that photos taken by individuals living with pain communicated their subjective experiences and highlighted their health concerns that may have been overlooked, unconceptualized, or ignored. This allowed these previously ignored concerns to be discussed and incorporated into their care plans. We offer that transforming the narratives of individuals in pain into more accessible forms of knowledge is important to consider clinically. Understanding the emotional dimensions of pain is the impetus of this level of engaged dissemination. Fraser and Sayah^26^ indicate that arts-based research is an effective method to discover, reﬂect, deliberate, and describe the art of nursing and medical care and argue that it is a more superior way of communicating the aesthetic component than the spoken word.

Others have employed the use of art in research to promote increased understanding of health and illness and argue that this creates a reframing whereby a clinician’s frame of reference shifts to that of the patient perspective.^8,41,53–55^ As argued by Lapum and colleagues,^41^ employing an arts-informed approach to research analysis and dissemination can potentially elicit affective responses, thus creating meaningful dialogue that engages clinicians at an embodied, emotive, and intellectual level. We suggest that arts-based research may provide an added reference that clinicians can use to assist in the assessment and evaluation of the multidimensional aspects of pain.

#### Strengths and Limitations

Employing the ABELE approach provided an aesthetic translation of patient narratives of their early ischemic cardiac pain and process of recognition of CAD. This integrated process involved qualitative analysis, literary interpretation through creation of thematic poetry, and additional representation through visual art. The ABELE approach may enhance the translation of patients’ health experiences with use in similar and/or different patient populations with acute, chronic, and palliative forms of pain or conditions.

Examining cardiac pain from a humanistic framework, brought to light through an arts-based qualitative level of inquiry, intentionally disrupts our notions of how we know and understand pain and switch our focus to what is important for patients regarding their multidimensional pain experiences. This form of artistic/scientific dissemination has the potential to transcend the barriers of language, to enable individuals to see, hear, speak, feel, and imagine what pain is like for an individual in the early stages of obstructive CAD.

This study captured the prodromal experiences of 23 men and women with CAD from only one location in southeastern Ontario. The prodromal pain-related symptoms and emotional journey as described by the participants may be different across other Canadian provinces and different countries and worthy of future investigation. Our sample was mostly homogeneous (Caucasian); a more diverse sample in terms of race, ethnicity, and marginalized/minority groups may add new information. Through no intention of the researchers, all artists who created the representations of thematic poetry were women. It would be prudent in subsequent arts-based studies of this nature to include a diverse representation by artists to include both genders and artists with multicultural backgrounds to ensure equal representation. More important, inclusion should also consider artists who have chronic pain or cardiac disease, which would richly add to the dynamic analysis.

#### Conclusion

Bridging the connection between science and art to disseminate the complex nature of living with cardiac-related prodromal pain and disease is important to consider clinically. When heart disease strikes, initial feelings of uncertainty, vulnerability, and fear are common. The jarring stories of women and men living through cardiac pain and recognition of CAD demanded a deeper level of analysis. The ABELE approach allowed our team to convey artistically the experience of living with prodromal pain in a powerful manner. Such arts-based representations challenge our assumptions by rendering the subjective pain experience and illness visible. This creates opportunity for mutual recognition between sufferer and viewer and may help to validate the emotional meaning of the pain experience.

## References

[1] Heart and Stroke Foundation. Women and heart disease and stroke. HSFC; 2018. http://www.heartandstroke.mb.ca/site/c.lgLSIVOyGpF/b.3661137/k.D95E/Heart_Disease__Women_and_heart_disease_and_stroke.htm.

[2] Bond S, Stonebridge C, Thériault L. The Canadian heart health strategy: risk factors and future cost implications. The Conference Board of Canada, Heart and Stroke Foundation of Canada and the Canadian Cardiovascular Society; 2010. https://sencanada.ca/content/sen/committee/412/SOCI/Briefs/2015-05-07ReportCdnCardiovascularSociety-AddInfoConferenceBoardofCanada_e.pdf

[3] Statistics Canada. Causes of death, Canada, 2013.Released 2014 1 28. https://www150.statcan.gc.ca/n1/pub/82-625-x/2017001/article/14776-eng.htm.

[4] Charles River Associates Life Sciences Practices. The economic and societal burden of acute coronary syndrome in Canada. 2010. http://www.crai.ca/sites/default/files/publications/Burden-of-acute-coronary-syndrome-in-Canada.pdf.

[5] O’Keefe-McCarthy S, McGillion M, Clarke S, McFetridge-Durdle J. Pain and anxiety in rural acute coronary syndrome patients awaiting diagnostic cardiac catheterization. J Cardiovas Nurs. 2014;30(6):546–57. doi:10.1097/JCN0000000000000203. 25325373

[6] Ploghaus A, Narain C, Beckmann CF, Clare S, Bantick S, Wise R, Matthews PM, Rawlins JN, Tracey I. Exacerbation of pain by anxiety is associated with activity in a hippocampal network. J Neuro. 2001;19:896–903. PMID: 11739597. 10.1523/JNEUROSCI.21-24-09896.2001 PMC6763058 11739597

[7] Rosen SD. From the heart to brain: the genesis and processing of cardiac pain. Can J Cardiol. 2012 Mar-Apr;28(2 Suppl):S7–S19. doi:10.1016/j.cjca.2011.09.010.S7-S19. 22424286

[8] McSweeney JC, Rosenfeld A, Abel WM, Braun L, Burke L, Burke LE, Daugherty SL, Fletcher GF, Gulati M, Mehta LS, et al. Preventing and experiencing ischemic heart disease as a woman: state of the science a scientific statement from the American heart association circulation. 2016, p. 1302–31. doi:10.1161/CIR.0000000000000381. PMC5154387 26927362

[9] O’Keefe-McCarthy S, Taplay K, Keeping-Burke L, Ostrowski L, Flynn-Bowman A, Vasilaki M, Vigo J, Hoelzli J, Salfi J, O’Leary D. Revision of the Cardiac Prodromal Symptoms-Screening Scale [PS-SS]: a qualitative exploration. CJCN. 2019;29:15–22.

[10] American Heart Association, Heart disease and stroke statistics-2014 update: a report from the American Heart Association, Circulation, 2014. Available from http:circ.ahajournals.org 10.1161/01.cir.0000441139.02102.80 PMC5408159 24352519

[11] Eftekhari H, Bukjarvoich I, Aziz E, Hong MK. Epidemiology and pathophysiology of acute coronary syndrome. In: Hong MK, Herzog E, editors. Acute coronary syndrome multidisciplinary and pathway-based approach. New York (NY): Springer; 2008. p. 25–36.

[12] Kumar A, Cannon CP. Acute coronary syndromes: diagnosis and management, part 1. Mayo Clin Proc. 2009;84(10):917–38. doi:10.1016/S0025-6196(11)60509-0. 19797781 PMC2755812

[13] Bahr R, Christenson R, Farin H, Hand M, Long JM. Prodromal symptoms of acute myocardial infarction: overview of evidence. Maryland Med. 2001;(Suppl):S49–S59. 11434061

[14] McSweeney JC, Cody M, O’Sullivan P, Elberson K, Moser DK, Garvin BJ. Women’s early warning symptoms of acute myocardial infarction. Circulation. 2003;108(21):2619–23. doi:10.1161/01.CIR.0000097116.29625.7C. 14597589

[15] O’Keefe-McCarthy S. Women’s experiences of cardiac pain: a review of the literature. CJCN. 2008;18(3):18–25. 18727283

[16] O’Keefe-McCarthy S, Ready L. Impact of prodromal symptoms on future adverse cardiac-related events: a systematic review. J Cardiovasc Nurs. 2016;31(1):E1–10. doi:10.1097/JCN.0000000000000207. 25419940

[17] Choinière M, Watt-Watson J, Victor JC; the CARD-PAIN Research Group (by alphabetical order): Baskett R.J.F., Bussières JS, Carrier M, Cogan J, Costello J, Feindel C, Guertin MC, Racine M, Taillefer MC. Prevalence and risk factors of persistent postoperative pain after cardiac surgery: CARD-PAIN – A 2-year prospective multicentre study. CMAJ. 2014;186(7):E213–23. doi:10.1503/cmaj.131012. 24566643 PMC3986330

[18] Katz J, Seltzer Z. Transition from acute to chronic postsurgical pain: risk factors and protective factors. Expert Rev Neurother. 2009;9(5):5,723–744. doi:10.1586/ern.09.20. 19402781

[19] Schwind JK. The narrative reflective process: giving voice to experiences of illness. In: McClean SL, editor. Creative arts in humane medicine. Edmonton (AB): Brush Education Inc. 2014. p. 125–40.

[20] Lapum J, Fredericks S, Beanlands H, McCay E, Scwhind JK, Romaniuk D. A cyborg ontology in health care: traversing into the liminal space between technology and person-centered practice. Nurs Phil. 2012;13(4):276–88. doi:10.1111/j.1466-769X.2012.00543.xMedline:22950731. 22950731

[21] Melzack R. Pain and the neuromatrix in the brain. J Dent Edu. 2001;655(12):1378–82. doi:10.1002/j.0022-0337.2001.65.12.tb03497.x. 11780656

[22] Melzack R, Wall P. Pain mechanisms: a new theory. Science. 1965;150(3699):971–79. doi:10.1126/science.150.3699.971. 5320816

[23] Charon R. The self-telling body. Narrative Inq. 2006;16(1):191–200. doi:10.1075/ni.16.1.24cha.

[24] Todres L, Galvin KT, Holloway I. The humanization of healthcare: a value framework for qualitative research. Intern J Qual Stud Health Well. 2009;4(2):68–77. doi:10.1080/174826208025=646204.

[25] Knowles JG, Cole AL. Handbook of the arts in qualitative research: perspectives methodologies, examples and issues. Los Angeles (CA): Sage; 2008.

[26] Fraser K, Sayah F. Arts-based methods in health research: a systematic review of the literature. Arts Health. 2011;3(2):110–45. doi:10.1080/17533015.2011.561357.

[27] Leavy P. Social research and creative arts: an introduction. In: Leavy P, editor. Method meets art-arts-based research practice. 2nd ed. New York (NY): Guildford Press; 2015. p. 1–38.

[28] Lapum J, Ruttonsha P, Church K, Yau T, Matthews DA. Employing the arts in research as an analytical tool and dissemination method: interpreting experience through the aesthetic. Qual Inq. 2012;18(1):100–15. doi:10.1177/1077800411427852.

[29] Stucky H, Tisdell E. The role of creative expression in diabetes: an exploration onto the meaning making process. Qual Health Res. 2010;20(1):42–56. doi:10.1177/1049732309355286. 19926796

[30] Leggo C. Astonishing silence: knowing in poetry. In: Knowles JG, Cole AL, editors. Handbook of the arts in qualitative research: perspectives, methodologies, examples, and issues. Los Angeles (CA): Sage; 2008. p. 165–74.

[31] Furman R, Collins K, Langer C, Bruce E. Inside a provider’s perspective: using practitioner poetry to explore the treatment of persons with mental illness. Arts Psychother. 2006;33(4):331–42. doi:10.1016/j.aip.2006.04.003.

[32] Bergold J, Thomas S. Participatory research methods: a methodological approach in motion. Forum: Qual Soc Res. 2012; 13(1):Art. 30. http://nbn-resolving.de/urn:nbn:de:0114-fqs1201302

[33] Heaton J. Focus: secondary analysis of qualitative data. Hist Soc Res. 2008;33:33–45.

[34] Sandelowski M. Focus on research methods. Whatever happened to qualitative description? Res Nurs Health. 2000;23(4):334–40. doi:10.1002/1098-240X(200008)23;4<334;;AID-NUR9><334;;AID-NUR9>3.0.C.0;2-G. 10940958

[35] Morgan DL. Focus group as qualitative research. 2nd ed. Thousand Oaks (CA): Sage; 1997.

[36] Miles M, Hubermann M. An expanded source book: qualitative data analysis. California (United States): Sage; 1994.

[37] Morse J, Field P. Qualitative research methods for health professionals. California (United States): Sage; 2005.

[38] Kvale S. Interviews: an introduction to qualitative research interviewing. California (United States): Sage; 1996.

[39] Pendergrast M. “Poem is what?” Poetic inquiry in qualitative social science research. Int Rev Qual Res. 2009;1(4):541–68. doi:10.1525/irqr.2009.1.4.541.

[40] Richardson L. The consequences of poetic representations: writing the other, writing the self. In: Ellis C, Flaherty MG, editors. Investigating subjectivity: research on lived experience. Newberry Park (CA): Sage; 1992. p. 125–37.

[41] Lapum J, Liu L, Church K, Hume S, Harding B, Wang S, Nguyen M, Cohen G, Yau TM. Knowledge translation capacity of arts-informed dissemination: a narrative study. Arts/Res Inter. 2016;1(1):258–82. doi:10.18432/R2BC7H.

[42] Henderson R. “Opening the Heart”: an Interview with Steve Parker. Psych Perspect. 2017;60(2):196–206. doi:10.1080/00332925.2017.1314700.

[43] Robinson KR. Developing a scale to measure denial levels of clients with actual or potential myocardial infarctions. Heart Lung. 1994;23:36–44. 8150643

[44] Perkins-Porras L, Whitehead DL, Strike P, Steptoe A. Prehospital delay in patients with acute coronary syndromes: factors associated with patient decision time and home to hospital delay. Eur J Cardiovasc Nurs. 2009;8(1):26–33. doi:10.1016/j.ejcnurse.2008.05.001. 18635400 PMC2652658

[45] Hart PL. Women’s perceptions of coronary heart disease: an integrative review. J Cardiovasc Nurs. 2005;20(3):170–76. doi:10.1097/00005082-200505000-00008. 15870587

[46] Isaksson RM, Brulin C, Eliasson M, Näslund U, Zingmark K. Older women’s prehospital experiences of their first myocardial infarction. J Cardiovasc Nurs. 2013;28(4):360–69. doi:10.1097/JCN.0b013e31824bcebc. 22495804

[47] McSweeney JC, Cleves MA, Zhao W, Lefler LL, Yang S. Cluster analysis of women’s prodromal and acute myocardial infarction symptoms by race and other characteristics. J Cardiovasc Nurs. 2010;25(4):311–22. doi:10.1097/JCN.0b013e3181cfba15. 20539165 PMC2884391

[48] Gallagher R, Marshall AP, Fisher MJ. Symptoms and treatment-seeking responses in women experiencing acute coronary syndrome for the first time. Heart Lung. 2010;39(6):477–84. doi:10.1016/j.hrtlng.2009.10.019. 20561851

[49] Padfield D. ‘Representing’ the pain of others. Health. 2011;15(3):241–57. doi:10.1177/1363459310397974. 21593050

[50] Padfield D, Zakrzewska JM, de Williams AC. Do photographic images of pain improve communication during pain consultations? Pain Res Manag. 2015;20(3):123–28. doi:10.1155/2015/145964. 25996763 PMC4447153

[51] Kenny DT. Constructions of chronic pain in doctor-patient relationships: bridging the communication chasm. Patient Ed Couns. 2004;52(3):297–305. doi:10.1016/S0738-3991(03)00105-8. 14998600

[52] Baker T, Wang C. Photovoice: use of a participatory action research method to explore the chronic pain experience in older adults. Qual Health Res. 2006;16(10):1405–13. doi:10.1177/1049732306294118. 17079801

[53] Colantonio A, Kontos P, Gilbert J, Rossiter K, Gray J, Keightley M. After the crash: research-based theatre for knowledge transfer. J Contin Edu Health Prof. 2008;28(3):180–85. doi:10.1002/chp.177. 18712795

[54] Elliott C, Elliott B. From the patient’s point of view: medical ethics and the moral imagination. J Med Ethics. 1991 12;17(4):173–78. doi:10.1136/jme.17.4.173. 1787514 PMC1376050

[55] Mitchell G, Dupuis S, Jones-Simpson C. Countering stigma with understanding. The role of theatre in social change and transformation. Can Theatre Rev. 2011;146(22):22–27. doi:10.3138/ctr.146.22.

